# Asymmetric response of different functional insect groups to low‐grazing pressure in Eurasian steppe in Ningxia

**DOI:** 10.1002/ece3.4611

**Published:** 2018-11-17

**Authors:** Zihua Zhao, Jing Wei, Kaiyang Zhang, Hao Li, Shuhua Wei, Xubin Pan, Wenguang Huang, Mengmeng Zhu, Rong Zhang

**Affiliations:** ^1^ Department of Entomology, College of Plant Protection China Agricultural University Beijing China; ^2^ Institute of Plant Protection Ningxia Academy of Agriculture and Forestry Yinchuan China; ^3^ Institute of Plant Quarantine Chinese Academy of Inspection and Quarantine Beijing China; ^4^ Grassland Station of Ningxia Yinchuan China

**Keywords:** community, diversity, ecological function, population density, predator

## Abstract

In recent years, the continued loss and fragmentation of steppe has caused decreased ecosystem functions and species losses in insect diversity. In the 2000s, the Chinese government developed a series of national projects, such as the construction of enclosures, to conserve natural ecosystems, including steppe. However, the effects of these enclosures on steppe arthropod community are largely unknown. In the present study, we selected enclosed and low‐grazing regions at eight National Grassland Fixed Monitoring Stations to examine the compositional differences in four insect functional groups and their associated ecological functions. The results showed that diversity significantly differed between the enclosed and low‐grazing regions, with the number of insect families being significantly higher in enclosed regions than in regions with low‐grazing pressure. The responses of the insect community to steppe management also varied among the four groups (herbivores, predators, parasitoids, and pollinators). The abundances of herbivores, predators, and parasitoids were higher in enclosed regions than in low‐grazing regions, while there was no significant difference in pollinators. Additionally, there were no significant differences in the predator/prey ratio between enclosed regions and low‐grazing regions in any of the steppe types. The parasitic wasp/prey ratio was higher in enclosed regions than in low‐grazing regions in meadow steppe and typical steppe, while there were no significant differences between the enclosed and low‐grazing regions in desert steppe and steppe desert. Herbivores were observed to benefit much more from enclosures than predators, parasitoids, and pollinators. Therefore, we recommend low‐grazing should be considered in steppe conservation, which could conserve biodiversity and achieve biocontrol functions of arthropod community.

## 1 INTRODUCTION

Steppe is an important habitat type in northwest China, harboring a highly diverse insect community and threatened ecosystems (Korosi et al., 2014; Tropek, Hejda, Kadlec, & Spitzer, 2013). However, the continued loss and fragmentation of natural habitats has caused the degeneration of steppe and associated ecosystem function in the past few decades, which has resulted in substantial concern around the world (Schindler et al., 2016; Shang et al., 2014). Furthermore, the changes in landscape pattern (land cover conversion) have caused severe biodiversity loss in steppe habitats across China (He, Liu, Tian, & Ma, 2014; Zhao, Sandhu, Ouyang, & Ge, 2016). On the one hand, the increasing demand for grains and vegetables has led to the rapid expansion of arable land, which occupies a large amount of steppe and increasing natural and semi‐natural habitats (Katayama, Osawa, Amano, & Kusumoto, 2015; Queiroz, Beilin, Folke, & Lindborg, 2014). On the other hand, the abandonment of poor arable lands and marginal lands often results in the biological invasion of small bushes or other alien plants, which directly causes secondary succession in natural steppe (Dengler, Janisova, Torok, & Wellstein, 2014; Vitkova, Muellerova, Sadlo, Pergl, & Pysek, 2017).

In the 2000s, the Chinese government developed a series of national projects to conserve natural ecosystems, including steppe (Hua & Squires, 2015). In northwest China, both fencing and grazing restriction strategies have been conducted to restore steppe ecosystems through the “Tianbao” project (Hao et al., 2014). Many enclosed areas have been established to enhance both insect and plant biodiversity (Marrero, Torretta, Vazquez, Hodara, & Medan, 2017; Mu, Zeng, Wu, Niklas, & Niu, 2016), and the plant diversity associated with aboveground net primary productivity (ANPP) has been greatly enhanced as a result. The insect fauna is also an important component in steppe, accounting for approximately 60% of all living species (plants 15% and vertebrates 4%) (Paschetta et al., 2013). Insects are characterized by high diversity due to their immense species richness and various life forms, including herbivores, pollinators, parasitoids, and predators, making insect communities an important part of terrestrial ecosystems, especially in steppe (Jackson, Turner, & Pearson, 2014; Schirmel, Bundschuh, Entling, Kowarik, & Buchholz, 2016). For example, herbivores could shift the plant community composition by feeding on different components of the plant community and disturbing interspecific relationships and can even affect the reproductive success of native plants (Franklin et al., 2016; Kaarlejarvi & Olofsson, 2014). Additionally, pollinators can benefit plants and enhance seed dispersal through the mutualistic interactions between these insects and the plants they pollinate (LeVan & Holway, 2015).

Until now, the effects of fencing and grazing prevention (based on enclosure strategies) on insect communities have been largely unknown (Reid, Fernandez‐Gimenez, & Galvin, 2014), and the response of insect communities to fencing and the prevention of grazing appears to vary at different spatial scales (Charles, Porensky, Riginos, Veblen, & Young, 2017). At the local scale, the enclosure of steppe causes changes in soil quality and microenvironments, which mediate the composition of plants associated with the invertebrate community (Macdonald et al., 2015; Schirmel et al., 2016). At the landscape scale, fencing and grazing prevention resulting from landscape simplification often negatively affect diversity and the abundance of various taxonomic groups, such as invertebrates (Kormann et al., 2015).

Ecological processes, the species pool, and diversity patterns depend on habitat composition, microenvironments, and landscape patterns (Alhamad & Alrababah, 2013; Seifert, Leuschner, & Culmsee, 2015). In particular, plant community associated with the landscape matrix could affect the mobility of organisms, which could also influence the structure of the insect community (Bezemer, Harvey, & Cronin, 2014; Marini et al., 2014). Thus, exploring the effects of ecological restoration measures on insect biodiversity and determining how to develop conservation strategies to enhance ecological functions are key topics in ecological conservation and reconstruction (Dietl et al., 2015; Mijangos, Pacioni, Spencer, & Craig, 2015).

In northwest China, steppe and shrub steppe are the most species‐rich ecosystems and can be divided into several main steppe types (Seabloom et al., 2013; Zhao & Li, 2013). At present, the steppes have evolved into climax communities that are also facing several challenges under global change (Frei, Ghazoul, Matter, Heggli, & Pluess, 2014; Lavergne, Mouquet, Thuiller, & Ronce, 2010). One of the most prevalent disturbances in steppe is livestock grazing, which can change plant community composition, soil compactness, and nutrient cycling (Andres et al., 2016; Elwell, Griswold, & Elle, 2016). In the past, overstocking was very common in China due to the increasing demand for production (Hou et al., 2014).

Grazers can also indirectly impact other grassland organisms, such as invertebrates and birds, through structural changes to the habitat caused by frequent herbivory and trampling (Sharps, Smart, Skov, Garbutt, & Hiddink, 2015). Many invertebrate groups have critical ecosystem functions in steppe ecosystems, and plants benefit from many of them through pollination and seed dispersal by insects (van Klink, Plas, Noordwijk, WallisDeVries, & Olff, 2015). The predators and parasitic wasps that attack herbivores and pollinators could make the plant–insect interactions more complex (Hamback, Inouye, Andersson, & Underwood, 2014). However, invertebrates, particularly pollinators, have been given less attention in grazing studies, especially in northwest China (Fantinato et al., 2016).

Many strategies (fencing and grazing prevention) have been developed to restore the vegetation cover and soil structure and recover steppe health (Zhao et al., 2017). Across the steppe of Ningxia, fencing, reduced grazing, and reseeding have been applied to restore ecosystem health in steppe (Chen, Wang, Zhou, Liu, & Huang, 2014). In addition, conservation areas (fully enclosed areas) have been established in different steppe types to improve steppe biodiversity in China. Recent research has shown that conservation strategies could effectively enhance plant diversity and the abundance of endangered species (Beever et al., 2016; Nagendra et al., 2013). In terms of invertebrate functional groups, there have been few experiments exploring the interaction between the conservation strategy used and the insect community (Senapathi, Goddard, Kunin, & Baldock, 2017). Therefore, based on the above literature, two questions were addressed: (a) Does the conservation strategy used in Chinese steppe (enclosures) increase the diversity and richness of the insect community in different steppe types? (b) Could the different functional groups of the insect community associated with different ecological functioning be enhanced under this conservation strategy compared with control conditions?

## 2 MATERIALS & METHODS

### 2.1 Study area

The study area was located in Ningxia Hui Autonomous Region of northwest China, which was a part of in Eurasian Steppe. Totally, there were four steppe types (meadow steppe (MS), typical steppe (TS), desert steppe (DS), and steppe desert (*SD*)) in Ningxia, which were widely distributed in Eurasian Steppe (Bai et al., 2008; Zhao et al., 2018). The four steppe types accounted for more than 90% of total steppe in Ningxia, which also had significant differences of plant biomass and species composition (see Supporting Information Table S1). A huge ecological restoration project was conducted in different steppe types during 2012–2015, which included establishment of the eight National Grassland Fixed Monitoring Stations (Nanhuashan, Guyuan, Zhangjiayuan, Zhongning, Hongsipu, Lingwu, Yanchi, and Zhongwei).

**Figure 1:**
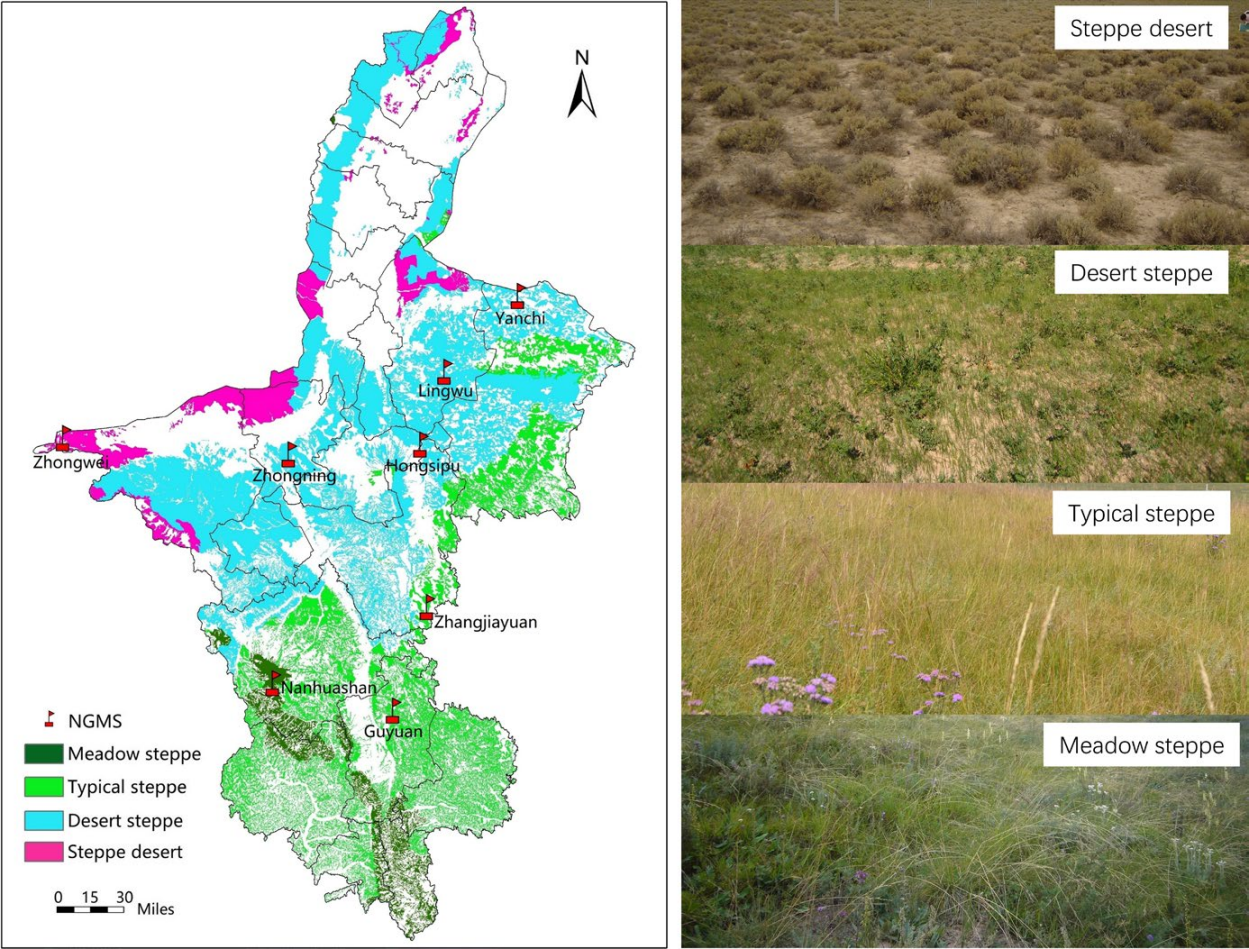
The map of Ningxia steppe, Northwest China (NGMS, the national grassland fixed monitoring stations, the different steppe types were indicated by different color. The 8 NGMS (Nanhuashan station, Guyuan station, Zhangjiayuan station, Yanchi station, Lingwu station, Hongsipu station, Zhongning station, and Zhongwei station) were indicated by red flags)

Nanhuashan station (105.6299E, 36.4052N) occurs in meadow steppe (MS) and was established in 2013. Guyuan station (106.2968E, 36.2803N) and Zhangjiayuan station (106.4955E, 36.7393N) occur in typical steppe (TS) and were established in 2013 and 2014, respectively, while Yanchi station (107.0476E, 38.0808N), Lingwu station (106.6201E, 37.7601N), Hongsipu station (106.4745E, 37.4393N), and Zhongning station (105.7266E, 37.4010N) occur in desert steppe (DS) and were established in 2012, 2014, 2015, and 2015, respectively. Finally, Zhongwei station (104.4476E, 37.4757N) occurs in steppe desert (*SD*) and was established in 2015. These eight national grassland fixed monitoring stations (NGMS) were established for the long‐term monitoring of the plant and insect communities (Figure 1).

A chain‐link fence (an iron net and pillar) was used to seal each NGMS, forming a completely enclosed or fenced region (~3 ha), and no livestock or other large herbivores were allowed to enter the enclosed areas. Regions with low‐grazing pressure (~1 individuals/ha/year) during May to October each year occur adjacent to each NGMS, while no grazing occurs in the other months.

### 2.2 Insect collection

Sticky traps (yellow) were used to collect insect samples in the studied regions. Five cards were placed at each NGMS to capture insects using a 5‐point sampling method, which is an empirical method used for insect collection (Zhao, Hui, Li, & Li, 2015). Each point was a replicate, and there were five replicates within each NGMS. The same 5‐point sampling method was used to collect insect samples in the adjacent regions with low‐grazing pressure. The sticky cards were randomly placed throughout the enclosed and low‐grazing regions for 5 days, following which all sticky cards were transported back to the laboratory for insect identification. The collection periods were 20–25 July in 2016 and 20–25 July in 2017 at all sampling locations.

### 2.3 Statistical analysis

The number of insect individuals captured in the field was counted for each card, and the mean values ± SE were then calculated. Based on family level, Shannon–Wiener index $\left(H=-\sum_{i=1}^{k}\left(P_{i}\right)\left(l_{n}P_{i}\right)\right)$ was used to compute the diversity of insect arthropods in four steppe types, respectively (Zhao et al., 2018). The insects were then classified into four functional groups (herbivore, pollinator, predator, and parasitic wasp). For each functional group, multiple comparisons and tests of the insect community across the two different treatments (enclosure regions and low‐grazing regions) and four different steppe types were examined to identify significant differences.

We conducted split‐plot analysis, as our designed experiments have different treatments applied to plots of different sizes. The steppe types were treatments, and sampling points within each steppe type were replicates. Mixed‐effects models were used to facility to deal with complicated error structures and hence avoid the pitfalls of pseudoreplication (Crawley, 2012). The function *lme* is called because the explanatory variables are a mixture of fixed effects (management treatment: enclosure regions and low‐grazing regions) and random effects (steppe types). All analyses were performed using the statistical software R 3.3.1 (R Development Core Team, 2016) with the “vegan” and “lmer” packages.

## 3 RESULTS

**Figure 2:**
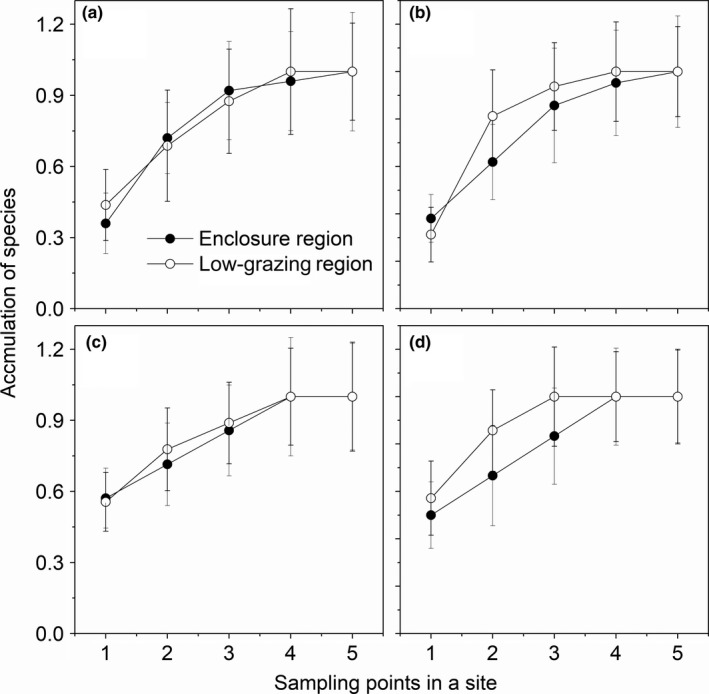
Species accumulation of sampling points in a site in enclosure regions (ER) and low‐grazing regions (LGR) four steppe types (a) meadow steppe; (b) typical steppe; (c) desert steppe; and (d) steppe desert. Solid circles are enclosure regions, and empty circles are low‐grazing regions

Three sticky traps (sampling points) of both enclosure and low‐grazing regions in meadow steppe could cover more than 85% species and four sticky traps could account for more than 95% species (Figure 2a). Additionally, three sticky traps could also include 89%, 87%, and 91% species in typical steppe, desert steppe, and steppe desert, respectively (Figure 2b–d). Additionally, more than 95% species could be collected by four sticky traps in all steppe types (Figure 2).

**Figure 3:**
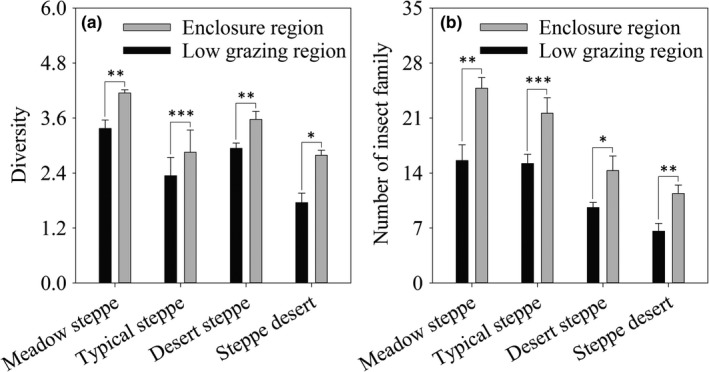
Insect community response (diversity (a) and number of insect families (b)) to different management patterns (enclosures and low‐grazing pressure) in four steppe types (meadow steppe, typical steppe, desert steppe, and steppe desert) in Ningxia, northwest China. Asterisks above the bars indicate differences between enclosure regions (ER) and low‐grazing regions (LGR) (^***^ *p* < 0.001; ^**^ *p* < 0.01; ^*^ *p* < 0.05). Black columns are enclosure regions, and white columns are low‐grazing regions

The responses of different groups to the enclosure regions (ER) and low‐grazing regions (LGR) were varied due to species‐specific (Table 1). Diversity significantly differed between the enclosure regions (ER) and low‐grazing regions (LGR) (*t*_1,9_ = 6.59, *p* = 0.006, Figure 3a) in meadow steppe. The ER also had higher diversity than that in LGR in typical steppe (*t*_1,19_ = 8.37, *p* < 0.001), desert steppe (*t*_1,39_ = 3.76, *p* = 0.01), and steppe desert (*t*_1,9_ = 4.09, *p* = 0.01, Figure 3a). Similarly, the number of insect families in ER in meadow steppe, typical steppe, desert steppe, and steppe desert was higher than that in LGR (MS, *t*_1,9_ = 9.68, *p* < 0.001; TS, *t*_1,19_ = 7.35, *p* < 0.001; DS, *t*_1,39_ = 2.68, *p* = 0.03; *SD*,* t*_1,9_ = 4.95, *p* = 0.01, Figure 3b).

**Table 1:** Mixed linear analysis (enclosures and low‐grazing pressure) of the main families of the insect community in different steppe types (meadow steppe, typical steppe, desert steppe, and steppe desert)

Groups and family	Meadow steppe		Typical steppe		Desert steppe		Steppe desert	
	*t*_1,9_	*p*	*t*_1,19_	*p*	*t*_1,39_	*p*	*t*_1,9_	*p*
Herbivores								
Chrysomelidae	2.92	0.05	0.71	0.53	3.84	0.01	2.21	0.10
Pyralidae	0.26	0.84	0.58	0.61	3.38	0.01	0.60	0.61
Noctuidae	0.44	0.71	1.35	0.23	3.27	0.01	1.69	0.18
Aphididae	1.42	0.25	1.36	0.22	2.93	0.02	—	—
Cicadellidae	2.12	0.11	0.10	0.95	1.71	0.14	—	—
Predators								
Syrphidae	0.18	0.90	0.30	0.81	0.16	0.91	1.21	0.32
Coccinellidae	3.24	0.04	0.71	0.53	1.23	0.24	1.46	0.24
Asilidae	1.55	0.22	1.03	0.35	0.32	0.78	1.13	0.35
Reduviidae	0.24	0.86	3.62	0.01	1.46	0.20	—	—
Chrysopidae	3.32	0.03	2.16	0.07	10.97	<0.001	—	—
Parasitoid wasps								
Ichneumonidae	2.41	0.08	2.88	0.02	1.20	0.29	8.53	0.00
Braconidae	2.12	0.11	0.10	0.95	1.66	0.14	3.79	0.02
Pteromalidae	2.04	0.12	0.73	0.51	0.95	0.39	4.86	0.01
Pollinators								
Apidae	1.10	0.41	2.03	0.11	1.64	0.18	—	—
Vespidae	0.89	0.51	2.38	0.06	1.26	0.19	0.85	0.52
Sphecidae	1.10	0.41	0.67	0.61	0.61	0.60	2.34	0.11

**Figure 4:**
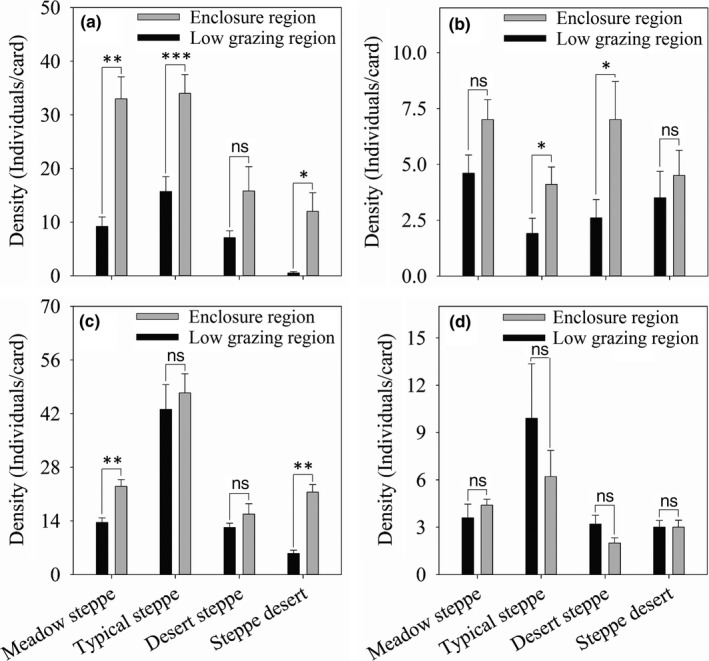
The abundances of different functional groups under different management patterns (enclosures and low‐grazing pressure) in four steppe types (meadow steppe, typical steppe, desert steppe, and steppe desert) in Ningxia, northwest China ((a), herbivores; (b), predators; (c), parasitoid wasps; (d), pollinators). Asterisks above the bars indicate differences in mean values among different steppe types (^***^ *p* < 0.001; ^**^ *p* < 0.01; ^*^ *p* < 0.05). Black columns are enclosure regions, and white columns are low‐grazing regions

Herbivore abundance in the ER of meadow steppe, typical steppe, and steppe desert was higher than that in LGR (MS, *t*_1,9_ = 2.09, *p* = 0.09; TS, *t*_1,19_ = 10.21, *p* < 0.001; *SD*,* t*_1,39_ = 3.92, *p* = 0.01, Figure 4a). However, there was no significant difference in the abundance of herbivores between ER and LGR in desert steppe (*t*_1,9_ = 2.35, *p* = 0.07, Figure 4a).

In terms of the other functional groups, the abundance of predators in the ER of typical steppe and desert steppe was higher than that in LGR (TS, *t*_1,19_ = 4.29, *p* = 0.04; DS, *t*_1,39_ = 6.28, *p* = 0.012, Figure 4b), while there was no significant difference in the abundance of predators between ER and LGR in both meadow steppe and steppe desert (MS, *t*_1,9_ = 1.39, *p* = 0.14; *SD*,* t*_1,9_ = 1.16, *p* = 0.38, Figure 4b). The abundance of parasitoid wasps in the ER of meadow steppe and steppe desert was significantly higher than that in LGR (MS, *t*_1,9_ = 5.29, *p* = 0.01; *SD*,* t*_1,9_ = 8.92, *p* = 0.002, Figure 4c), while there was no significant difference in the other two steppe types (typical steppe and desert steppe) (TS, *t*_1,19_ = 1.08, *p* = 0.36; DS, *t*_1,39_ = 0.68, *p* = 0.78, Figure 4c). There were no differences in pollinator abundance between ER and LGR in any of the steppe types (MS, *t*_1,9_ = 0.69, *p* = 0.67; TS, *t*_1,19_ = 0.43, *p* = 0.91; DS, *t*_1,39_ = 1.24, *F* = 0.38; *SD*,* t*_1,9_ = 0.68, *p* = 0.62, Figure 4d).

**Figure 5:**
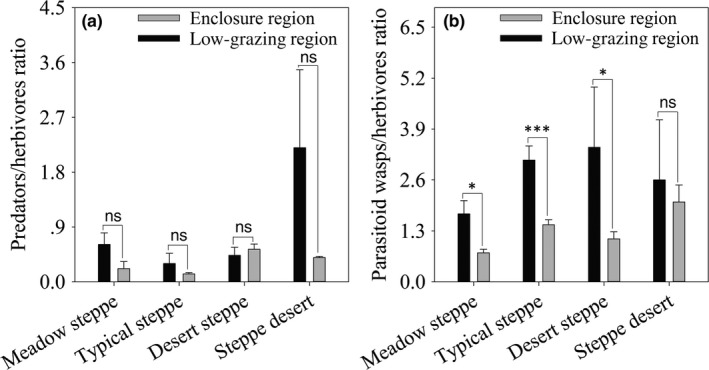
Effects of different management patterns (enclosures and low‐grazing pressure) on the biocontrol function (predator/herbivore ratio (a) and parasitoid wasp/herbivore ratio (b)) in different steppe types (meadow steppe, typical steppe, desert steppe, and steppe desert) in Ningxia, northwest China. Asterisks above the bars indicate differences in mean values among the different steppe types (^***^ *p* < 0.001; ^**^ *p* < 0.01; ^*^ *p* < 0.05). Black columns are enclosure regions, and white columns are low‐grazing regions

The predator/herbivore ratio in the ER of all steppe types was not significantly different from that in LGR (MS, *t*_1,9_ = 2.38, *p* = 0.09; TS, *t*_1,19_ = 1.67, *p* = 0.62; DS, *t*_1,39_ = 1.38, *p* = 0.59; *SD*,* t*_1,9_ = 1.53, *p* = 0.28, Figure 5a). In contrast, the parasitoid wasp/herbivore ratio in LGR in meadow steppe, typical steppe, and desert steppe was significantly higher than that in ER (MS, *t*_1,9_ = 3.92, *p* = 0.02; TS, *t*_1,19_ = 6.92, *p* < 0.001; DS, *t*_1,39_ = 3.24, *p* = 0.04), while there was no significant difference in steppe desert (*t*_1,9_ = 0.42, *p* = 0.79, Figure 5b).

## 4 DISCUSSION

Since the middle of the 20th century, a series of changes has occurred in steppe use in China: (a) large‐scale landscape modification of natural environments with changes in land cover, (b) afforestation of “bare” lands, (c) abandonment of infertile arable lands, and (d) overstocking (Ambarli et al., 2016). To face the challenges of biodiversity loss and ecosystem degradation under global change, many conservation strategies have been implemented to restore ecosystems. In general, the abundance and diversity of insects could be influenced by the intensity of management (enclosures), especially in grasslands (Newbold et al., 2015; Vitousek, 1994). However, insect richness was found to be largely unaffected by land use intensity (grazing and mowing frequency) across several groups (Swengel, 2001). Simons et al. (2017) also found that the intensity of land use affected the taxonomic richness of only plants and herbivores, while grazing intensity affected the taxonomic richness of all groups (Simons et al., 2017).

Unmanaged steppe could enhance the abundance and diversity of Orthoptera assemblages (herbivores) compared with managed grasslands in Mediterranean steppe rangeland (Alignan, Debras, & Dutoit, 2014). However, Goodenough and Sharp (2016) also found that moderate grazing intensity in both autumn and winter could enhance the abundance of butterflies while having disadvantageous effects on plants in winter (Goodenough & Sharp, 2016). McIver and Macke (2014) even found an increase in the species richness and abundance of the butterfly community in steppe after artificial disturbances (fire or mechanical treatments) (McIver & Macke, 2014). Therefore, low‐grazing pressure and disturbance could facilitate most insect taxa while having no effects on other species (Lazaro et al., 2016). Light grazing resulted in larger local populations of butterflies compared to heavy grazing or no grazing at all (Johansson, Knape, & Franzen, 2017); thus, it is possible to considerably reverse the negative trends and reduce extinction risk through conservation actions. Furthermore, the abundance and richness of herbivores could be greatly increased through effective enclosure strategies, which was well supported in our present experiment.

For predators and parasitic wasps, there have been fewer experiments examining the effects of grazing on natural enemy richness or diversity. Weking, Kampf, Mathar, and Holzel (2016) reported that the abundance and diversity of herbivores (Orthoptera) could be enhanced by grazing across western Siberia (Weking et al., 2016). However, different functional groups of cursorial spiders (Aranei) and true bugs (Heteroptera) in northeastern Ukraine had varied responses to management intensity via the gully terrain (slope or bottom) (Polchaninova, Savchenko, Drogvalenko, Ronkin, & Shabanov, 2016). In our experiment, the abundance of predators was higher in ER than in LGR only in typical and desert steppe, while the abundance of parasitic wasps was higher in ER than in LGR only in meadow and steppe desert. Therefore, different functional groups have different responses to the management pattern, and the nature of these responses depends on species‐specific characteristics. Benitez‐Lopez, Vinuela, Mougeot, and Garcia (2017) found that low levels of management (the rotation of plowing and fallows and a reduction in the frequency and intensity of plowing) could benefit sandgrouses (steppe birds) and other steppe species, while both leaving land fallow (no disturbance) and highly intense agriculture (arable lands) have detrimental effects on bird conservation (Benitez‐Lopez et al., 2017).

In our experiment, we found that pollinators showed no significant response to unmanaged steppe, which indicates that a complete enclosure strategy could not effectively conserve pollinators. Klink et al. (2016) found that low stocking densities favored high abundances of voles, pollinators, and flowers (van Klink, Plas, Noordwijk, WallisDeVries, & Olff, 2016). However, the bird community showed no significant responses to the grazing level (Howland et al., 2016). Biocontrol functions (predator/herbivore and parasitic wasp/herbivore ratios) were not also enhanced by the enclosure strategy in the present experiment, which indicates that complete enclosures can impede the sustainable management of steppe. The varied responses of different groups to the management pattern in steppe were an important reason for this phenomenon. In general, herbivores benefited more from the enclosures than their natural enemies (predator and parasitic wasps). The homogeneous vegetation structure in the enclosed regions may not be attractive to predators (ladybeetles) or parasitic wasps (Aphidiidae), which have been reported to be sensitive to the management activity in steppe (Schachat et al., 2014). In contrast, low‐grazing pressure caused patchy vegetation cover, including areas containing different plant species or puddles (Simons et al., 2017). The abundance of some insects, including dung beetles and flies, could be increased by the feces of grazing animals (Beynon, Wainwright, & Christie, 2015). Grazing can indirectly enhance biodiversity via changing vegetation cover and hence improve biocontrol functions in regions with low‐grazing pressure (van Klink et al., 2015). Therefore, different steppe use patterns have district effects on different insect functional groups and need to be considered separately when studying the effects of steppe use on ecological communities (Macdonald et al., 2015). Additionally, the use of common and rare species as additional parameters describing the overall composition of the insect community will shed light on the potential mechanisms behind the effects of different steppe use patterns (Simons et al., 2017).

## 5 CONCLUSION

Enclosures in the Ningxia steppe enhanced the diversity and number of insect families. However, the responses of different insect functional groups to the enclosures varied due to their varied feeding characteristics and other species‐specific factors. Enclosures could increase the abundance of herbivores while having no effect on pollinators. Furthermore, full enclosures reduced the parasitoid wasp/herbivore ratio and impeded the service of biocontrol. Biocontrol functions can be greatly enhanced in steppe by optimizing grassland utility via grazing intensity. Conservation measures that are focused on enclosures cannot achieve the aim of biodiversity conservation (Kormann et al., 2015). Therefore, light grazing should be considered on increase biocontrol functions have been newly considered to conserve biodiversity and achieve sustainable management (Jennings, Smith, Fulton, & Smith, 2014; Weking et al., 2016).

## CONFLICT OF INTEREST

None declared.

## AUTHORS CONTRIBUTIONS

Z Zhao and R Zhang designed the experiments. Z Zhao, S Wei, Mg Zhu, and W Huang performed the experiments and collected the data in the field. Z Zhao analyzed the data and wrote the first draft. Z Zhao, J Wei, K Zhang, H Li, X Pan, and R Zhang revised the manuscript and approved the final version.

## DATA ACCESSIBILITY

Insect species composition in enclosure and low‐grazing regions of steppe: Dryad https://doi.org/10.5061/dryad.50sk900.
