# Supplementary material for: Asymmetric response of different functional insect groups to low‐grazing pressure in Eurasian steppe in Ningxia
Source: Ecol Evol. 2018 Nov 17;8(23):11609–18. doi: 10.1002/ece3.4611 (PMC6303718; doi:10.1002/ece3.4611)
Supplement: Supplementary file 1 [file ECE3-8-11609-s001.docx]

**Table S1** The detailed descriptor of 4 steppe types in Ningxia

| Steppe types | Detailed descriptors |
| --- | --- |
| Meadow steppe | Meadow steppe is mainly distributed in South Ningxia (Liupan mountain and Nanhua mountain), and comprises about 88,700 ha. Yearly precipitation in meadow steppe ranges from 350 to 450mm and the effective accumulated temperature (≥10 °C) ranges from 1800 to 2000 day degrees. In meadow steppe, grass height is 60–80 cm and ground cover is 60–85%, while the ANPP per ha is 3375–6000 kg. |
| Typical steppe | Typical steppe is mainly distributed in Yunwu mountain and Yueliang mountain, comprising about 635,920 ha. Yearly precipitation in typical steppe ranges from 250 to 450mm and grass height is 30–50 cm. The effective accumulated temperature (≥10 °C) in typical steppe is 1600–1800 day degrees. |
| Desert steppe | Desert steppe, which is the dominant steppe type (accounting for 1,443,030 ha) in China, is mainly found in Xiang mountain and Yanchi. Yearly precipitation in desert steppe is below 200mm and the main vegetation is drought grass and shrub. The effective accumulated temperature (≥10 °C) in typical steppe is 1500–1700 day degrees. |
| Steppe Desert | Steppe desert is mainly found in central Ningxia (Zhongwei), and accounts for about 226, 970 ha. Yearly precipitation in steppe desert is 100–150mm and the main vegetation is strong drought shrub and semi-shrub. |
